# Effect of corneal cross-linking on endothelial cell density and morphology in the peripheral cornea

**DOI:** 10.1186/s12886-020-01415-y

**Published:** 2020-04-07

**Authors:** Hiroyasu Goukon, Kazutaka Kamiya, Masahide Takahashi, Nobuyuki Shoji

**Affiliations:** 1 Department of Ophthalmology, School of Medicine, Kitasato University, Kanagawa, Japan; 2 Visual Physiology, School of Allied Health Sciences, Kitasato University, 1-15-1 Kitasato, Sagamihara, Kanagawa 2520373 Japan

**Keywords:** Corneal cross-linking, CXL, Endothelial cell density, Coefficient of variation, Hexagonal cells

## Abstract

**Background:**

To compare the endothelial cell density and morphology in the peripheral cornea before and after **c**orneal cross-linking (CXL).

**Methods:**

This study evaluated thirty-one eyes of 31 patients who were treated with standard CXL for progressive keratoconus. Preoperatively and 6 months postoperatively, we compared the corneal endothelial cell density (ECD), the coefficient of variation in cell size (CV), and the percentage of hexagonal cells (HEX), in the peripheral regions of the cornea, using a non-contact specular microscope (EM-3000, Tomey).

**Results:**

All keratoconic eyes in this series were measurable in the peripheral regions. No significant differences were found in the peripheral ECD preoperatively and 6 months postoperatively at each point (Wilcoxon signed-rank test, superior, *p* = 0.16, nasal superior, *p* = 0.12, temporal superior, *p* = 0.17, inferior, *p* = 0.37, nasal inferior, *p* = 0.28, temporal inferior, *p* = 0.17). The mean percentage of the ECD loss was 1.3, 1.3, 1.0, 1.4, 0.7, and 1.4%, respectively. No significant differences in the peripheral CV or HEX were found preoperatively and 6 months postoperatively at each point.

**Conclusions:**

Standard CXL does not cause significant changes in endothelial cell density, polymegethism, or polymorphism, in the peripheral regions of the cornea. It is suggested that CXL is a minimally invasive surgical approach for progressive keratoconus, even in terms of peripheral endothelial cells.

**Trial registration:**

University Hospital Medical Information Network Clinical Trial Registry (000031162).

## Background

Corneal cross-linking (CXL) has been acknowledged as an effective method for halting the progression of keratoconus by photosensitization of riboflavin activated by ultraviolet-A (UV-A) [1–6]. For this surgical indication, it is usually recommended that the preoperative corneal thickness is at least 400 μm, in order to prevent corneal endothelial cell damage caused by UV-A irradiation [7–9]. It has been reported that even 5 years after CXL, the endothelial cell density (ECD) did not change significantly at any postoperative interval [10]. It is sometimes difficult to determine the ECD in the central cornea in post-CXL eyes with progressive keratoconus, possibly as a result of the presence of apical scarring and/or a steep curvature of the cornea. Therefore, it is possible that the ECD measurements in the central cornea are less accurate than that in the peripheral cornea. Also, the standard CXL treatment requires a UV-A irradiation of 8- to 9-mm in diameter. Therefore, this treatment may cause changes in the corneal endothelial cell density and morphology, in both the central area and the peripheral area. To the best of our knowledge, no quantitative study has been performed on the changes in the peripheral corneal endothelial cells in post-CXL eyes. This study aims to assess the changes in the peripheral corneal endothelial cell density and morphology in eyes having standard CXL treatment for progressive keratoconus.

## Methods

### Study population

We registered the study protocol with the University Hospital Medical Information Network Clinical Trial Registry (000031162). We enrolled thirty-one eyes of 31 patients (12 men and 19 women) in this retrospective study. The sample size in this study offered 92.0% statistical power at the 5% level in order to detect a 100-cells/mm^2^ difference in the ECD, when the standard deviation (SD) of the mean difference was 160 cells/mm^2^. All patients received standard CXL treatment for progressive keratoconus at Kitasato university hospital, and completed a 6-month follow-up. The patients were recruited in a continuous cohort. Diagnosis of keratoconus was performed by one experienced clinician (K.K.) with evident findings characteristic of keratoconus (e.g., corneal topography with asymmetric bow-tie pattern with or without skewed axes), and at least one keratoconus sign (e.g., stromal thinning, conical protrusion of the cornea at the apex, Fleischer ring, Vogt striae, or anterior stromal scar) on slit-lamp examination [3]. Progression of the disease was interpreted as an increase in the maximum keratometric reading of at least 1 diopter (D), or a deterioration of corrected visual acuity with an increase of astigmatism ≥1 D confirmed in at least 2 examinations during the preceding 6 to 12 months before treatment. Due to safety issues related to corneal endothelial cells, CXL was not done in eyes with thinner corneas (when the thinnest corneal thickness is less than 400 μm). We excluded eyes with pellucid marginal degeneration, other corneal diseases, and previous ocular trauma or surgery. We received written informed consent for the CXL treatment from all patients. This clinical chart review was approved by the Institutional Review Board of Kitasato University, and followed the tenets of the Declaration of Helsinki. Our Institutional Review Board waived the requirement for informed consent for this retrospective review. The data that support the findings of the present study are available from the corresponding author upon reasonable request.

### Assessment of corneal endothelial cells

Preoperatively and 6 months postoperatively, we quantitatively evaluated the ECD, the coefficient of variation in cell size (CV), and the percentage of hexagonal cells (HEX), in the peripheral regions (2, 4, 6, 8, 10, and 12-o’clock positions on a 6-mm arc) of the cornea, using a non-contact specular microscope (EM-3000™, Tomey, Aichi, Japan). Patients were asked to fixate at each peripheral target, and photographs were taken of the peripheral corneal endothelium using the auto-alignment function. The system automatically measures the area of corneal endothelial cells (> 100 cells), and after that it automatically calculates the ECD, CV, and HEX within an area of 0.1 mm^2^ by the instrument’s built-in software for cell analysis.

### Surgical procedures of corneal cross-linking

The standard CXL technique was performed in accordance with the Dresden protocol [1]. Topical anesthesia was administered, and the corneal epithelium at a central circular area of 8-mm in diameter was removed with a blunt spatula. Next, we topically administered riboflavin 0.1% solution every 2 min for 30 min, and we used a slit-lamp microscope to confirm that adequate riboflavin had penetrated into the anterior chamber. UVA irradiation at a wavelength of 370 nm and a surface irradiance of 3 mW/cm^2^ was performed for 30 min with an OptoXLink corneal cross-linking system™ (North Miami, FL, US). Riboflavin solution was administered every 5 min during the irradiation. We used topical steroidal and antibiotic medications 4 times daily for 2 weeks after treatment, with the dose being reduced gradually thereafter, with a soft contact lens until re-epithelialization.

### Statistical analysis

All statistical analyses were performed with a commercially available statistical software (Bellcurve for Excel, Social Survey Research Information Co, Tokyo, Japan). In order to compare the pre- and the post-surgical data in the peripheral regions, we performed the Wilcoxon signed-rank test. The results are shown as mean ± SD, and a value of *p* < 0.05 was considered statistically significant, unless otherwise indicated.

## Results

The preoperative patient demographics are summarized in Table 1. The results of the peripheral ECD, CV and HEX before and after CXL are summarized in Table 2. We did not find any definite intraoperative complications. All eyes in this series were measurable in the peripheral regions of the cornea.

**Table 1:** Preoperative demographics of the study population

Demographic	Mean ± standard deviation (range)
Age (years)	26.2 ± 8.6 (20 to 47)
Gender (% female)	61
Spherical equivalent refraction (D)	−4.52 ± 4.28 (1.00 to −13.50)
Manifest cylinder (D)	− 4.19 ± 3.79 (0.00 to − 11.00)
UDVA (logMAR)	0.25 ± 0.27 (0.7 to − 0.08)
Mean keratometric readings (D)	49.8 ± 3.2 (44.2 to 55.3)
Central cornea thickness (μm)	478.1 ± 51.1 (400 to 581)
Stage of keratoconus (eyes)	
Stage I	8
Stage II	18
Stage III	5

*D* Diopter, *LogMAR* Logarithm of the minimal angle of resolution, *CDVA* Corrected distance visual acuity

**Table 2:** Endothelial cell density, coefficient of variation, and percentage of hexagonal cells in the peripheral regions of the cornea, before and after operation of corneal crosslinking (CXL)

Location	ECD (cells/mm^2^)			CV (%)			HEX (%)		
	Preoperative	Postoperative (6 months)	*P* value	Preoperative	Postoperative (6 months)	P value	Preoperative	Postoperative (6 months)	P value
Superior	2843 ± 271	2803 ± 293	0.16	41.3 ± 5.7	42.5 ± 6.5	0.23	34.9 ± 10.7	33.8 ± 9.9	0.14
Nasal Superior	2815 ± 297	2772 ± 273	0.12	41.2 ± 5.4	42.4 ± 6.8	0.21	35.7 ± 8.2	35.7 ± 9.0	0.80
Temporal Superior	2749 ± 264	2716 ± 245	0.17	42.1 ± 6.3	43.2 ± 6.7	0.11	33.4 ± 10.7	33.0 ± 10.8	0.30
Inferior	2759 ± 281	2734 ± 227	0.28	41.6 ± 6.5	42.5 ± 6.9	0.21	32.5 ± 8.5	33.0 ± 9.6	0.38
Nasal Inferior	2703 ± 452	2655 ± 645	0.37	41.6 ± 5.0	42.8 ± 7.6	0.15	31.4 ± 8.9	31.2 ± 10.1	0.49
Temporal Inferior	2645 ± 390	2603 ± 491	0.17	42.0 ± 7.3	42.9 ± 7.4	0.19	32.0 ± 8.7	31.9 ± 10.1	0.64

*ECD* Endothelial cell density, *CV* Coefficient of variation, *HEX* percentage of hexagonal cells

### Endothelial cell density

**Fig. 1:**
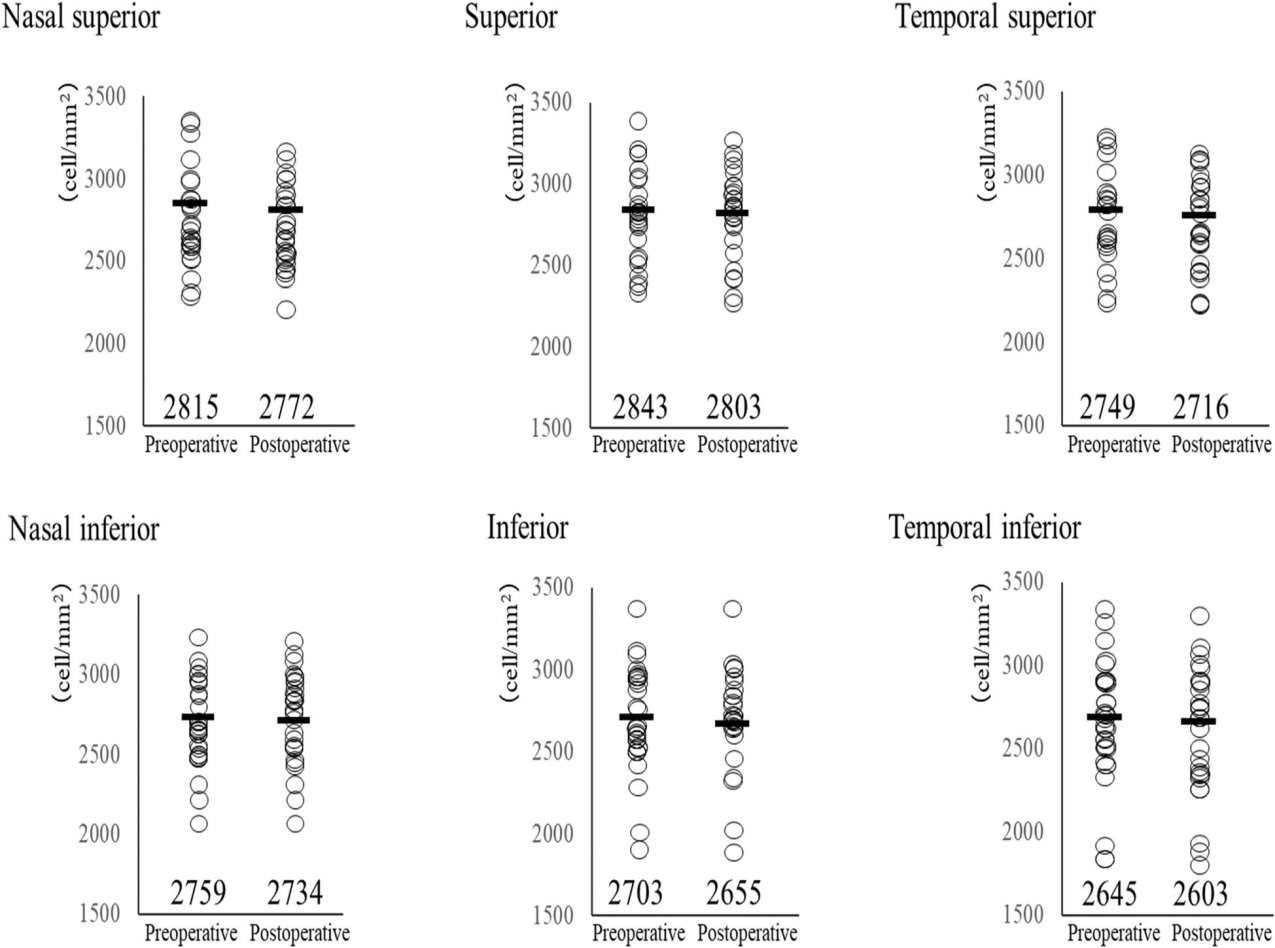
Endothelial cell density (ECD) in the peripheral regions before and 6 months after cross-linking (CXL)

The preoperative and postoperative ECD in the peripheral regions are shown in Fig. 1. No significant differences at each point were found in the peripheral ECD preoperatively and 6 months postoperatively (Wilcoxon signed-rank test, superior, *p* = 0.16, nasal superior, *p* = 0.12, temporal superior, *p* = 0.17, inferior, *p* = 0.37, nasal inferior, *p* = 0.28, temporal inferior, *p* = 0.17). The mean percentage of the ECD loss was 1.3, 1.3, 1.0, 1.4, 0.7, and 1.4%, respectively.

### Coefficient of variation

**Fig. 2:**
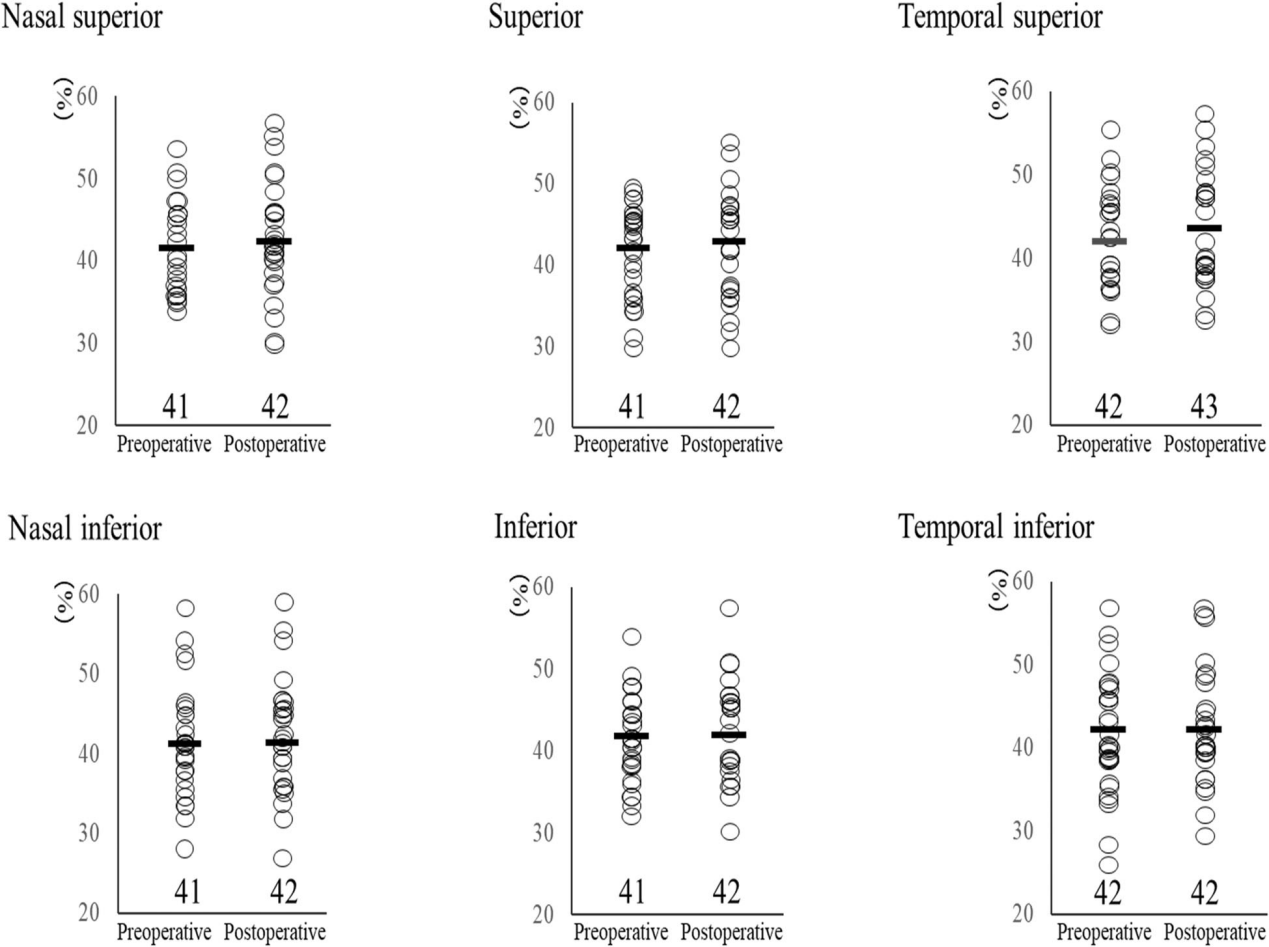
Coefficient of variation (CV) in the peripheral regions before and 6 months after corneal cross-linking (CXL)

The preoperative and postoperative CV in the peripheral regions are shown in Fig. 2. No significant differences were found at each point in the peripheral CV preoperatively and 6 months postoperatively (superior, *p* = 0.23, nasal superior, *p* = 0.21, temporal superior, *p* = 0.11, inferior, *p* = 0.15, nasal inferior, p = 0.21, temporal inferior, *p* = 0.19).

### Percentage of hexagonal cells

**Fig. 3:**
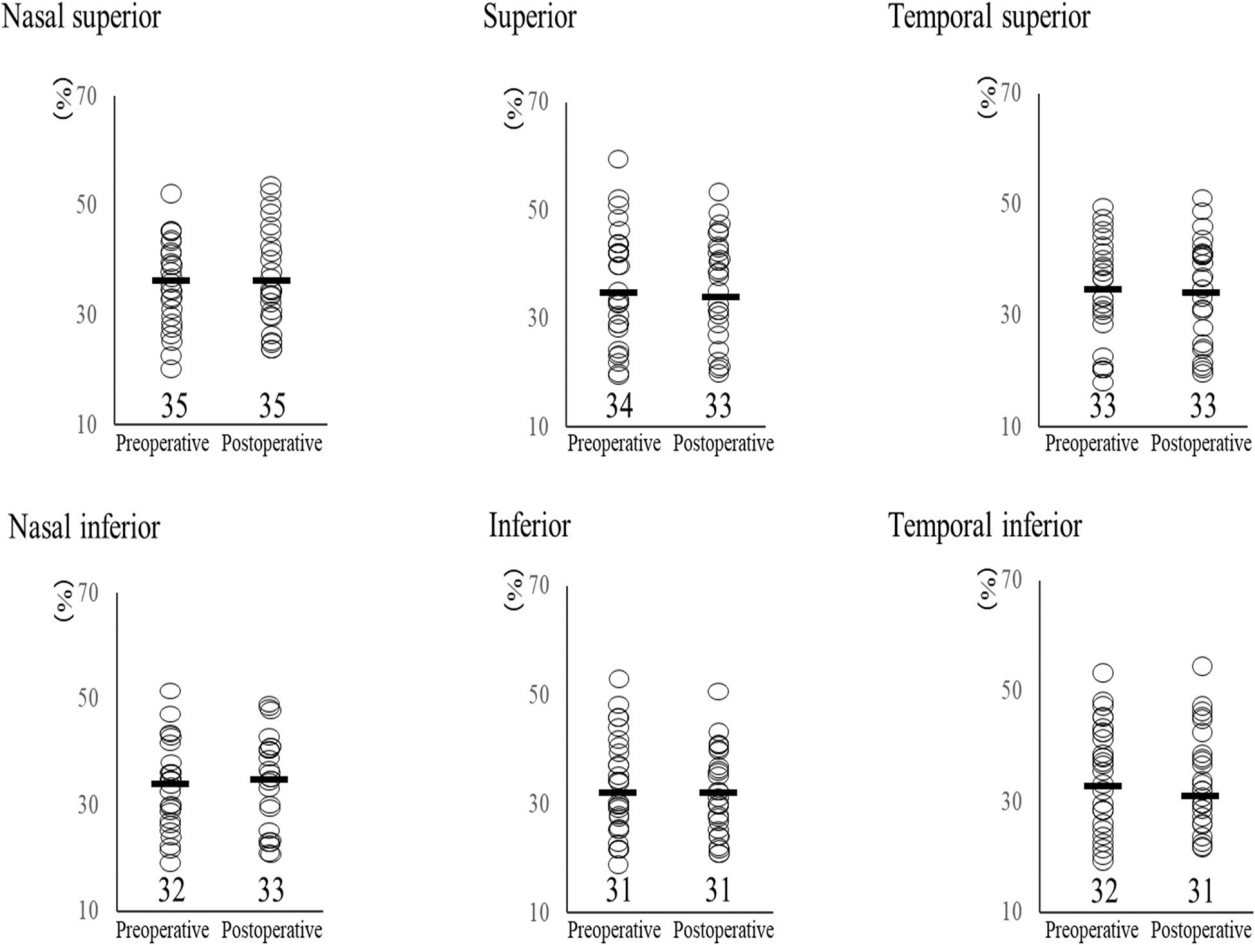
Percentage of hexagonal cells (HEX) in the peripheral regions before and 6 months after corneal cross-linking (CXL)

The preoperative and postoperative HEX in the peripheral regions are shown in Fig. 3. No significant differences were found at each point in the peripheral HEX preoperatively and 6 months postoperatively (superior, *p* = 0.14, nasal superior, *p* = 0.80, temporal superior, *p* = 0.30, inferior, *p* = 0.49, nasal inferior, *p* = 0.38, temporal inferior, *p* = 0.64).

## Discussion

The results of this study showed that the mean peripheral ECD loss was 0.7 to 1.4% 6 months after CXL, and that there were no significant changes in the peripheral ECD before and after CXL. This indicates that the standard CXL treatment was safe in terms of the peripheral ECD. This study also demonstrated that there were no significant changes in corneal morphological parameters such as CV or HEX, indicating that the CXL treatment did not cause a significant change in peripheral polymegethism or polymorphism. To the best of our knowledge, this is the first study on the detailed analysis of the peripheral endothelial cell density and morphology before and after CXL treatment.

Table 3 is a summary of previous studies on the long-term changes in the central ECD (spanning more than 2 years), and those on the changes in the central CV and HEX after CXL. Several studies on corneal endothelial cell after CXL have been published, however all studies focused on the endothelial cell density and the morphology only in the central cornea in post-CXL eyes [10–21]. The mean central ECD loss was − 0.1 to 7.5%, which was largely different among previous studies. We speculate that it is still challenging to exactly determine the ECD in some eyes with progressive keratoconus, as a result of the presence of apical scarring and/or a steep curvature of the cornea. With the exception of two studies, the peripheral ECD loss in our study almost matched the central ECD in previous studies [11, 16]. It should be noted that we cannot directly compare the ECD data after CXL, since the sample size, the observation period, the surgical technique, the location, and the measurement devices were different among the current and previous studies. With regard to the distributions of the peripheral ECD, our findings were in accordance with previous findings, in that the ECD in the superior region was slightly higher than that in the other regions [22].

**Table 3:** Previous studies on the long-term (spanning more than 2 years) changes in the central ECD loss, and those on the changes in the central CV and HEX after CXL

Author	Year	Eyes	Follow-up	Surgical Method	ECD loss (%)	CV, HEX (%)	Location	Device
Vinciguerra P et al. [11]	2009	28	2Y	Standard	4.9	N.A.	Center	Unknown **(**Konan)
Caporossi A et al. [12]	2010	44	4Y	Standard	0.7	N.A.		Noncon ROBO V **(**Konan)
Spada L et al. [13]	2012	16	1Y	Trans-epithelial	0.5	CV 28 to 29		SP-500 **(**SEED)
Kymionis GD et al. [10]	2014	25	5Y	Standard	3.6	N.A.		EM-3000 (TOMEY)
Wittig-silva C et al. [14]	2014	46	3Y	Standard	2.5	N.A.		SP-2000 (Topcon)
Cingü AK et al. [15]	2014	36	6 M	Accelerated	0.7	CV 44 to 46		Noncon ROBO SP-6000 (Konan)
						HEX 45 to 44		
Goldich Y et al. [16]	2014	17	3Y	Standard	7.5	N.A.		Noncon ROBO SP-6000 (Konan)
Nasrollahi K et al. [17]	2015	140	1Y	Standard	1.5	CV 39 to 40		Unknown (TOMEY)
						HEX 54 to 53		
Sedaghat M et al. [18]	2015	97	1Y	Standard	3.3	CV 18 to 20		SP-2000 (Topcon)
Sadoughi MM et al. [19]	2016	15	1Y	Standard	1.5	CV 34 to 35		SP-2000 (Topcon)
						HEX 56 to 57		
Giacomin NT et al. [20]	2016	40	4Y	Standard	−0.1	N.A.		Unknown **(**Konan)
Badawi AE et al. [21]	2016	40	1Y	Accelerated	0.9	CV 35 to 36		EM-3000 (TOMEY)
						HEX 55 to 56		
Current		31	6 M	Standard	1.2*	CV 41 to 42*	Periphery	EM-3000 (TOMEY)
						HEX 33 to 33*		

*M* Month, *Y* Year, *ECD* Endothelial cell density, *CV* Coefficient of variation, HEX = percentage of hexagonal cells, *CXL* Corneal crosslinking. *The average data of 6 peripheral measurement points

In this study, we found no significant changes in the peripheral CV or HEX after CXL, which was the same as previous studies with no significant changes in the central CV or HEX [13, 15, 17–19, 21]. The cellular stress levels of the cornea are chiefly reflected by the variations in the mosaic cell surface such as the presence of abnormally large cells and the deviation from their hexagonal shape. We previously reported that this device has provided good repeatability of the peripheral ECD, CV, and HEX measurements in eyes implanted with phakic intraocular lenses [23]. The differences in software for analyzing corneal endothelial cells, angle of view, and counting cells, and the reproducibility of the measurements may contribute to the discrepancy, especially for the CV and the HEX measurements. Therefore, the CV and HEX data should be interpreted with some caution and cannot be interchangeably compared among the devices.

There are several research limitations that affect this study. First, the study was conducted in a retrospective fashion. Second, the sample size was relatively small. Third, the follow-up period is up to 6 months. A further long-term prospective study with a large cohort of patients undergoing CXL is necessary to confirm our preliminary findings.

## Conclusions

In summary, our results support the view that the peripheral ECD, CV and HEX remained stable during the 6-month follow-up in post-CXL eyes. Based on the findings of this study, it is suggested that CXL did not cause a significant change in the density or the morphology of the peripheral corneal endothelial cells, and it is therefore a minimally invasive surgical approach, in terms of peripheral endothelial cells, for progressive keratoconus.

## Data Availability

The data that support the findings of the present study are available from the corresponding author upon reasonable request.

## Abbreviations

CXL: Corneal cross-linking
UV: ultraviolet
ECD: endothelial cell density
SD: standard deviation
D: diopter
CV: coefficient of variation
HEX: percentage of hexagonal cells
